# Flexible Photodetectors Based on 1D Inorganic Nanostructures

**DOI:** 10.1002/advs.201500287

**Published:** 2015-12-07

**Authors:** Zheng Lou, Guozhen Shen

**Affiliations:** ^1^ State Key Laboratory for Superlattices and MicrostructuresInstitute of SemiconductorsChinese Academy of SciencesBeijing100083P.R. China

**Keywords:** flexible electronics, photodetectors, nanowires

## Abstract

Flexible photodetectors with excellent flexibility, high mechanical stability and good detectivity, have attracted great research interest in recent years. 1D inorganic nanostructures provide a number of opportunities and capabilities for use in flexible photodetectors as they have unique geometry, good transparency, outstanding mechanical flexibility, and excellent electronic/optoelectronic properties. This article offers a comprehensive review of several types of flexible photodetectors based on 1D nanostructures from the past ten years, including flexible ultraviolet, visible, and infrared photodetectors. High‐performance organic‐inorganic hybrid photodetectors, as well as devices with 1D nanowire (NW) arrays, are also reviewed. Finally, new concepts of flexible photodetectors including piezophototronic, stretchable and self‐powered photodetectors are examined to showcase the future research in this exciting field.

## 1 Introduction

Flexible electronics devices that can be twisted, bent, stretched and folded have the potential for development for unique applications, such as wearable electronics, sensory skins, smart clothes, and implantable biomedical devices, etc, which cannot be achieved by existing rigid technology.^1^, ^2^, ^3^, ^4^ Several kinds of innovative flexible electronic devices have been successfully fabricated, such as flexible solar cells, electronic skins, flexible transistors and circuits, flexible light‐emitting diodes (LEDs), flexible batteries. and flexible photodetectors.^5^, ^6^, ^7^, ^8^, ^9^, ^10^, ^11^, ^12^, ^13^, ^14^, ^15^ Photodetectors (PDs) are a kind of electronic sensor for sensing light. PDs on rigid substrates have great applications in daily life like digital cameras, fire monitoring, and biological analysis, as well as military applications.^16^, ^17^, ^18^, ^19^, ^20^ Compared to PDs based on rigid substrates, flexible and soft devices can be better shaped and adapted to different surfaces which enables new functionalities such as being more implantable, allows for fewer aberrations, and better portability that cannot be fulfilled by their rigid counterparts.^21^, ^22^, ^23^, ^24^, ^25^, ^26^ For instance, flexible PDs could be attached to the human body so that one can easily detect ultraviolet radiation. Flexible PDs implanted on human eyes could help restore vision in blind people. Inspired by the interesting and novel and potential applications, great progress on flexible PDs has been obtained in the past several years, and many kinds of flexible PDs have been designed. For example, Rogers and his group developed a novel flexible PD based on single‐crystal silicon by compressible interconnects, and the device can improve imaging capability by mounting onto an eye‐ball lens.^27^

For most of the flexible devices, due to the basic physical properties of the plastic substrates the choice of the suitable sensing candidate material is a big challenge.^28^, ^29^ In the past, much attention has been paid to 1D semiconducting nanostructures, for example nanorods, nanowires (NWs), nanobelts and nanotubes, because their unique properties enable promising applications as functional components in nanoscale electronics and optoelectronics.^30^, ^31^, ^32^, ^33^, ^34^, ^35^, ^36^, ^37^, ^38^, ^39^, ^40^, ^41^ Due to the large surface to volume ratio, 1D nanostructures have a large number of surface trap states which can prolong the photocarrier lifetimes. Moreover, the low dimensionality of 1D nanostructures could shorten the transit times and limit the effective area of the charge carriers which lead to large responsivity and photoconductive gain in nanoscale PDs.^42^, ^43^, ^44^, ^45^, ^46^ In addition, compared with the other nanostructures, 1D nanostructures have the single dimensional characteristics, so it is easier to quantitatively analyze some key parameters of flexible devices such as deformation mode, deformation degree, bending stiffness, as well as possible crack characteristics.^47^ Furthermore, with their huge aspect ratios, 1D nanostructures are much more mechanically floppy along their natural growth direction. For a given bending radius, the wire‐like geometry will show great resilience to stress, and apparently, the deformation process hardly causes the crack formation laterally for its tiny size in diameter compared with other nanostructures. Therefore, due to these superior electrical properties and excellent mechanical flexibility, 1D inorganic nanostructures were extensively studied as building blocks for flexible PDs.

This article presents a comprehensive summary of 1D inorganic‐nanostructure‐based flexible PDs developed in the past. As there are many comprehensive reviews focusing on 1D inorganic nanostructure synthesis and assembly,^25^ this field will not be reviewed in this article. Three kinds of flexible PDs are first discussed in detail in this review, divided by their functionality, namely, flexible ultraviolet PDs, visible PDs, and infrared PDs. Next, the influence of organic–inorganic hybrid and 1D NWs arrays on their photoconductive properties and flexibilities would be presented. New concepts in flexible PD design and development are then introduced, followed by an outline of expectations for the future direction of research into 1D, inorganic‐nanostructure‐based, flexible PDs.

This is an open access article under the terms of the Creative Commons Attribution License, which permits use, distribution and reproduction in any medium, provided the original work is properly cited.

## 2 1D Inorganic‐Nanostructures‐Based Flexible PDs

The photoresponse of 1D inorganic nanostructures to light irradiation with different wavelengths is mainly depended on their band gap. For example, metal oxides such as ZnO,^48^ SnO_2_ 49 and In_2_O_3_,^50^ have large band gaps, thus their 1D nanostructures are widely used as the sensing candidates for high‐performance UV PDs. For visible light PDs, some semiconductors with moderate band gaps such as CdS,^51^ Si^52^ and In_2_Se_3_ 53 are chosen as the active materials. In_2_Te_3_ 54 and InAsSb,^55^ and semiconductors, with narrow band‐gaps may be used to fabricate infrared PDs. Here, recent progress of flexible, 1D, inorganic‐nanostructure‐based PDs are summarized, focusing on the above mentioned three types devices: UV PDs, visible PDs and infrared PDs.

### 2.1 Flexible Ultraviolet PDs

As an important type of optoelectronic device, UV PDs show wide ranging applications in fire monitoring, biological, environmental sensors, space exploration, and UV irradiation detection.^56^, ^57^, ^58^ High performance UV PDs have been widely developed based on 1D metal oxides or multicomponent metal oxides. In 2002, Yang et al. first reported single‐crystalline ZnO and SnO_2_ NWs based UV PD.^59^ The results revealed that the conductance of ZnO/SnO_2_ NWs changed greatly when the devices were exposed to ultraviolet light irradiation. Hence, great attention has been paid on developing high performance UV PDs with 1D metal oxide nanostructures, such as binary metal oxides, multicomponent metal oxides, and complex metal oxides as well.

#### 2.1.1 Flexible Binary‐Oxide‐Based PDs

**Figure 1:**
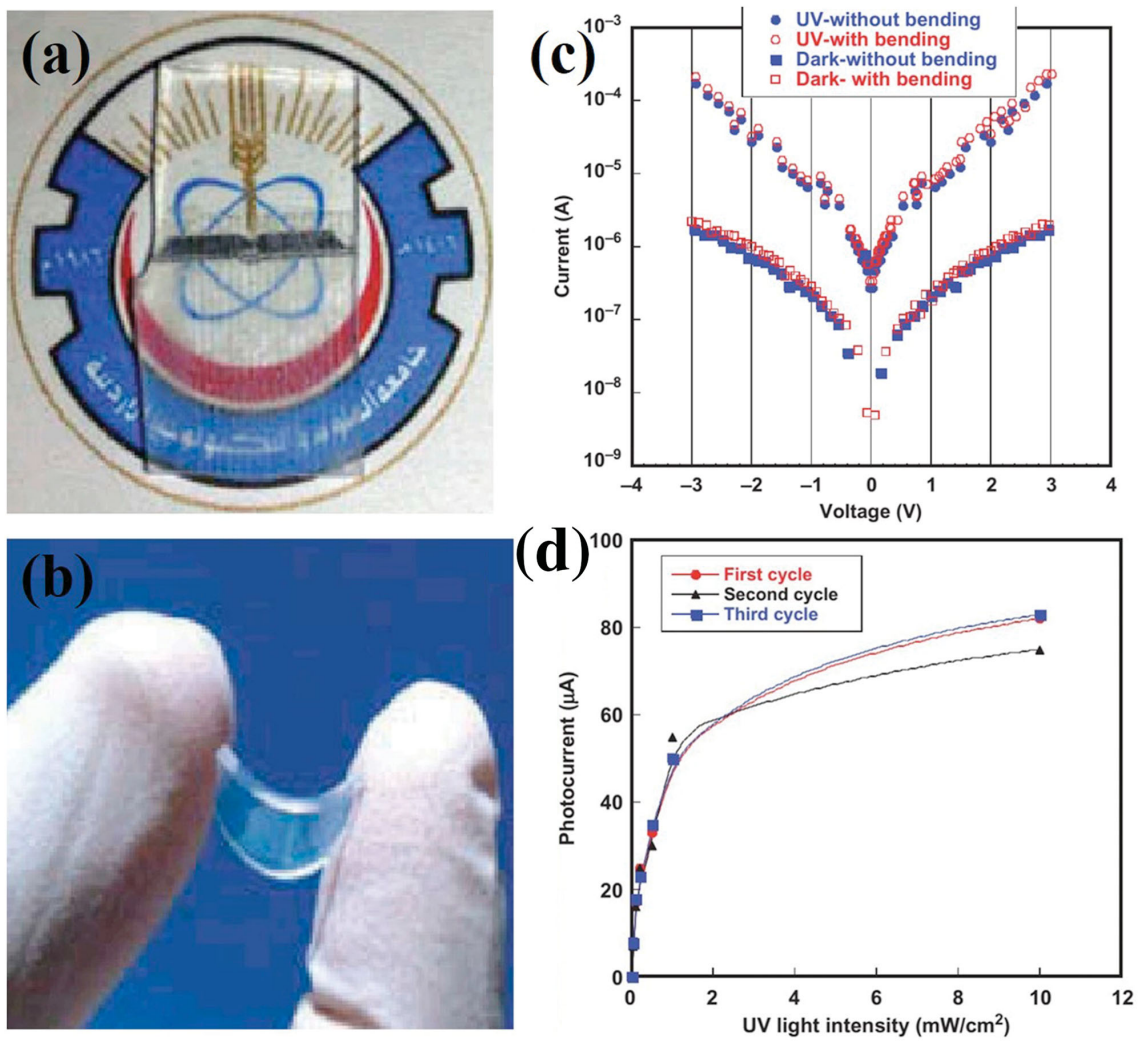
Flexible ZnO‐NR/PDMS UV PDs. a, b) Digital photographs of the flexible device, exhibiting excellent transparency and flexibility. c) *I–V* curves of the ZnO–NR/PDMS detector in darkness and in UV illumination (with and without bending). d) The response photocurrent at different UV light intensities. Reproduced with permission^66^ Copyright 2015, Taylor and Francis.

1D binary metal oxide nanostructures are good candidates for flexible UV PDs because of their excellent electronic properties, mechanical flexibility, and stability. Up to now, flexible UV PDs have been built on many kinds of 1D binary metal oxide nanostructures, including ZnO NWs/nanorods/nanobelts, SnO_2_ NWs/nanobelts, TiO_2_ nanorods, In_2_O_3_ NWs, etc.^60^, ^61^, ^62^, ^63^, ^64^, ^65^ An example is the flexible UV PDs developed by Albiss et al. They grew ZnO nanorods on a flexible polydimethylsiloxane substrate as shown in **Figure** **1**a,b; the device exhibited excellent transparency and flexibility.^66^ Figure 1c shows the current–voltage (*I–V*) properties of the device with and without bending when the device was exposed to 365 nm UV light and in the dark, respectively, while Figure 1d shows the photocurrent versus light intensities curves of the device under bending for different cycles. These results indicated that the flexible device based on ZnO nanorods has a good electronic stability and mechanical robustness.

Flexible UV PDs built on other 1D binary metal oxides nanostructures also exhibited attractive performances. For instance, a single SnO_2_ microrod UV photoconductor on a flexible substrate demonstrated excellent UV light selectivity and ultrahigh internal gain (1.5 × 10^9^), much higher than other SnO_2_ UV PDs.^67^ The findings in this research results in a novel and feasible method for realizing SnO_2_ microrod UV PD with both fast response speed and ultrahigh gain. In addition, a flexible UV PDs of ZnO NWs with 5–20 nm in diameter have also been fabricated using a UV decomposition process.^68^ The significant *I*_on_/*I*_off_ ratio of flexible device is about 11300% under 365 nm UV light. These studies indicate that 1D binary oxides have a great potential in flexible UV PDs.^69^, ^70^, ^71^

#### 2.1.2 Flexible Multicomponent‐Oxide‐Based PDs

**Figure 2:**
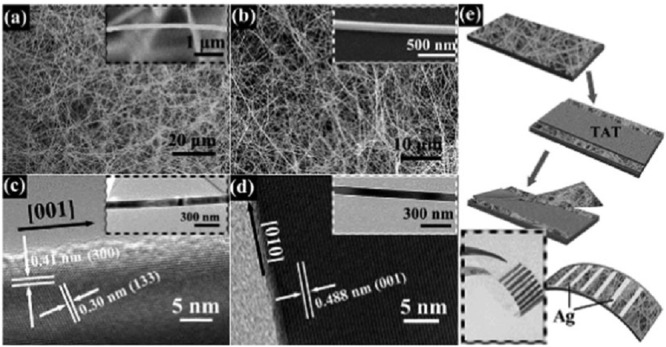
Flexible Zn_2_GeO_4_ and In_2_Ge_2_O_7_ NW networks based UV PDs. a,b) scanning electron microscope (SEM) images and c,d) transmission electron microscope (TEM) images of the Zn_2_GeO_4_ and In_2_Ge_2_O_7_ NWs, respectively. e) Schematic illustration of the fabrication process of flexible devices. Reproduced with permission.^78^ Copyright 2012, Optical Society of America.

Compared to simple binary oxides, multicomponent oxides exhibit superior performances, and functionalities of multicomponent oxides can be effectively tuned by altering the compositions.^72^, ^73^ Furthermore, most of the multicomponent oxides have wide‐bandgaps which make them essential components of a UV PD. 1D ternary oxides used in PDs, such as Zn_2_SnO_4_,^74^ In_2_Ge_2_O_7_,^75^ Zn_2_GeO_4_,^76^, ^77^ etc. have been investigated as functional materials for UV PDs. Flexible multicomponent‐oxide‐based PDs were first fabricated with Zn_2_GeO_4_ and In_2_Ge_2_O_7_ NW networks, as shown in **Figure** **2**.^78^ Zn_2_GeO_4_ and In_2_Ge_2_O_7_ NW networks were successfully prepared via a standard chemical vapor deposition (CVD) technology. After transferring the NWs to the flexible PET substrate, the silver paste were then printed as parallel lines. As‐fabricated flexible PDs exhibited outstanding photoresponse performance to UV light irradiation.

Individual single‐crystalline ZnGa_2_O_4_ NW was also investigated as the UV sensing material.^79^ Via a standard micro‐fabrication process, the single‐NW flexible PD was fabricated: ZnGa_2_O_4_ NWs were stripped and dissolved in isopropyl alcohol solution from the substrate, after that the NWs were dispersed on a PET substrate. The interdigitated electrodes (Cr/Au) were patterned at both ends of the NWs by photolithography techniques and lift‐off process. The flexible PD revealed a high response to 350 nm UV light illumination with an excellent photoresponse performance. The current on/off ratio is about 43 when the applied voltage is 5 V. The response and recovery times of the flexible ZnGa_2_O_4_ PDs are about 13 s and 9 s, respectively. The conductance of the device is hardly influenced by the bending stress, revealing the outstanding folding tolerance of the flexible ZnGa_2_O_4_ PD.

#### 2.1.3 Flexible Complex‐Structure‐based PDs

**Figure 3:**
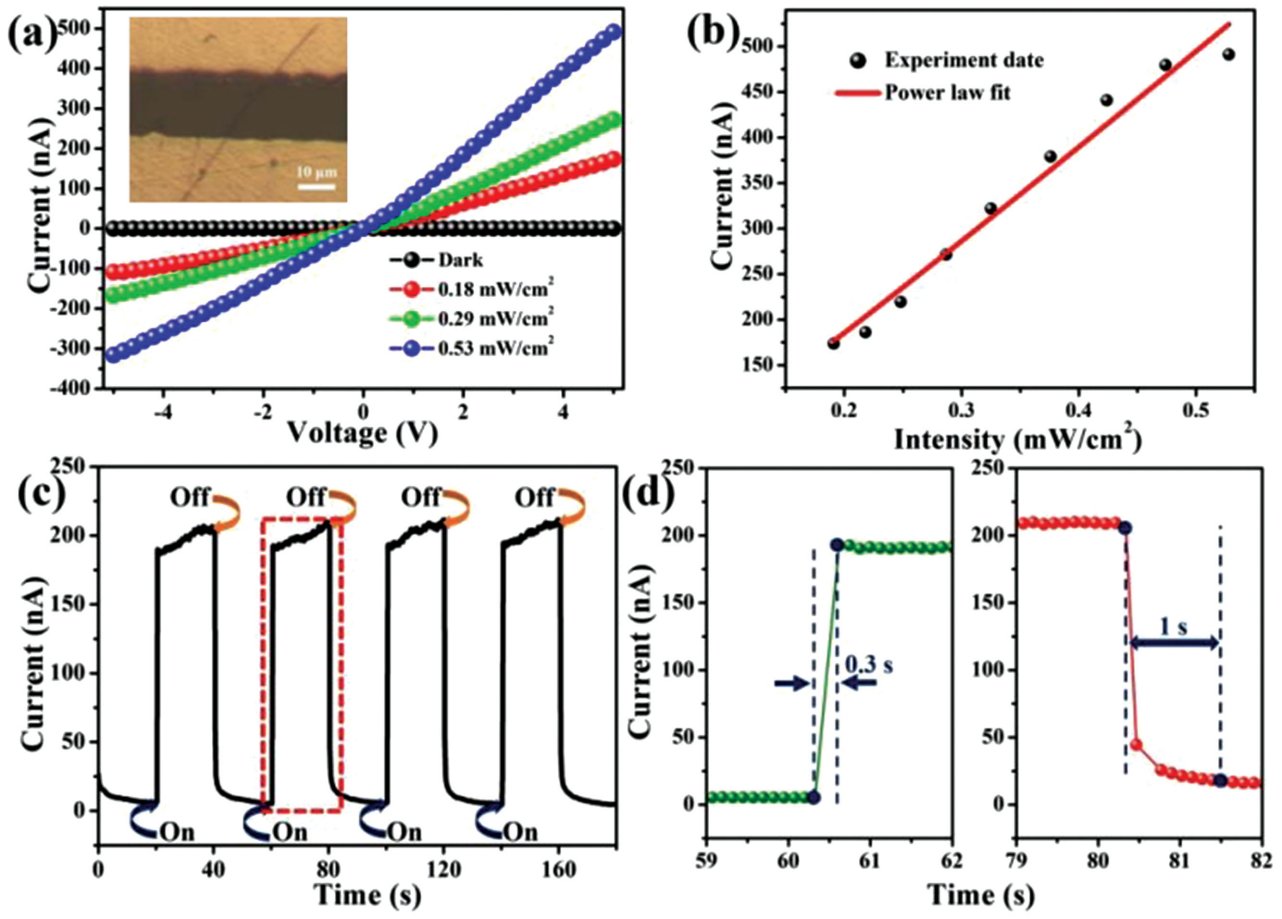
Flexible InGaO_3_(ZnO)‐superlattice‐NW‐based UV PDs. a) *I–V* test result of the device under different power intensities and in dark. b) Photocurrent versus light intensity. c) Photoresponse properties of the PD. d) Enlarged view of the middle cycle. Reproduced with permission.^81^

Some complex structures, such as heterostructures, and superlattice structures, have unique electronic and photonic properties with promising applications in optoelectronics. The determination of the characteristics of the materials with these structures are not only the collection of the contributions by each component, but also the reactions occurring between the various components of the interface.^80^ Recently, field‐effect transistors and flexible UV PDs were successfully fabricated with InGaO_3_(ZnO) superlattice NWs.^81^ The SEM image of the flexible device based on InGaO_3_(ZnO) superlattice NWs is shown in the inset of **Figure** **3**a. Figure 3a exhibited the *I–V* measurement of the flexible PD under a monochromatic UV light or in the dark. It can be seen that the device has a good Ohmic contact between the Cr/Au electrodes on the PET substrate and the superlattice NW. Figure 3b showed a good dependence between the photocurrent and the light intensity, which further proved a good photocapture in the superlattice NW. As shown in Figure 3c is the response and recovery properties of the device. During four testing cycles, the PD still keeps its original photocurrent, which further confirm that the as‐prepared flexible devices have good reproducibility. The influence of superlattice structure on the optoelectronic properties was proposed that recombination of the electron–hole pairs can be greatly decreased because of the spatial separation of the photogenerated carriers at the interface within the superlattice NWs. This work would be a reference for the superlattice NWs used in the field of PDs. Undoubtedly, more research works will focus on this field. Wei and his groups also reported a transparent UV PD based on the ZnO/SnO_2_ heterojunction nanofibers. Compared to pure SnO_2_ or ZnO nanostructures the ZnO/SnO_2_ device has a great enhancement of UV sensitivity due to the spatial of the photogenerated carriers.^82^ Similar results were also found in other research works, such as ZnS/ZnO,^83^ ZnO/graphene,^84^ etc.

### 2.2 Flexible Visible PDs

Due to the possibility of vast number commercial and military applications, visible light (400–780 nm) PDs have attracted great attention. A wide range of 1D inorganic semiconductor nanomaterials, including IV elements (Ge, Si),^85^, ^86^ III–V compounds (InP, GaAs),^87^, ^88^ II–VI compounds (CdS),^89^ group compounds (Cd_3_P_2_, Zn_3_As_2_)^90^, ^91^ and other compounds,^92^ have been applied to fabricate 1D‐nanostructure‐based visible light PDs.

#### 2.2.1 Flexible Silicon‐NW‐based PDs

**Figure 4:**
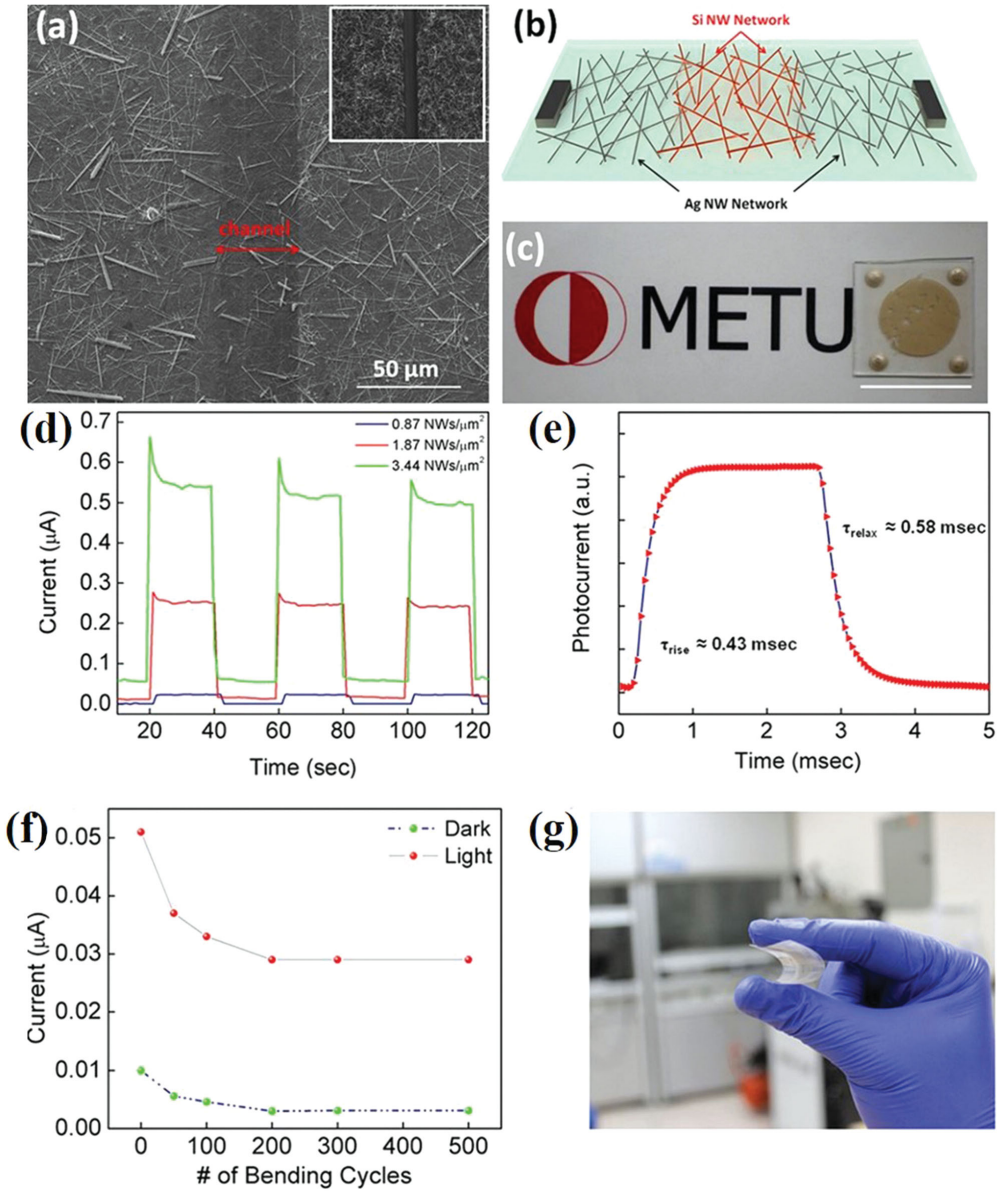
Flexible Si‐NW‐based PDs. a) SEM image of the MSM structure. Inset shows that the channel was free from Ag NW prior to Si NWs transfer. b) Representative device architecture and c) photograph of the final device. d) Light on–off measurements with different NW densities and e) dynamic response behavior. f) The stability of the devices in the investigated range. g) Photograph of flexible device at a bending radius of 1 cm. Reproduced with permission.^98^ Copyright 2013, American Institute of Physics.

Being the major material of the semiconductor industry, Si NWs have become one of the most emergent 1D materials which have been widely used in many application, such as solar cells,^93^ field effect transistors (FETs),^94^ thermoelectric applications,^95^ lithium batteries,^96^ and PDs.^97^ The use of Si NWs in PDs can enhance light harvesting and high quantum efficiencies, but the rigidity of the structure prevents flexible applications. Recently, the fabrication of highly flexible, Si NWs (via a metal‐assisted‐chemical etching method) network based PDs was studied by Unalan et al.^98^ They showed that the device provided both flexibility and transparency, because both the active part and the electrodes are composed by NWs networks. **Figure** **4**a is the SEM image of Ag and Si NW based metal–semiconductor–metal (MSM) PDs. Following mechanical scratching, the channel was free from Ag NWs (in the inset of Figure 4a). Figure 4b investigated the schematic of the devices in this work. A photograph of the finalized device (Figure 4c) reveal that the active area of the device was 3 mm. Photoresponsivity of the Si NW network PDs with different NW densities were also investigated. From the light on/off measurements, PDs were found to exhibit fully reversible switching behavior. As shown in Figure 4e, the typical rise time was found to be 0.43 ms, while the fall time was 0.58 ms. In order to measure the flexibility of the MSM PDs, the author fabricated the devices on PET substrate underwent a bending test. The photoconductor performance was recorded as a function of number of bending cycles for a fixed bending curvature of 1 cm (Figure 4f). The dark and photo current of the device measured as function of cycles up to a maximum bending cycles of 500 are shown in Figure 4g. Bending resulted in a decrease in both the light and dark current of the device during the first 200 cycles; however, further increase in the number of bending cycles did not lead to any change in the current. The main reason may be due to the loss of mechanical contacts between Si NW junctions and also Ag and Si NWs. This work provides the basis for the fabrication of cost‐effective and flexible PDs using Si NWs, which offer flexibility to the Si world and could certainly be helpful for the fabrication of other optoelectronic and sensing devices.

Si NWs can also be used in the flexible avalanche PDs (APDs). Kim et al. reported the fabrication of APDs composed by p^+^–i–n^+^ Si NWs on a flexible plastic substrate.^99^ The maximum responsivity and avalanche gain of the APDs are estimated to be 1.4 × 10^−3^ A/W and 5.9 × 10^4^, respectively. The superior photoresponse properties of the flexible APD are associated with the length of the intrinsic region of the NWs and the doping concentration. Furthermore, the APDs on the flexible plastic substrates showed excellent recovery properties as the device was bent and returned to the flat condition.

#### 2.2.2 Flexible II–V‐compound‐based PDs

II–V group semiconductors holds appeal for a diverse range of applications. Examples range from the highly earth‐abundant Zn_3_P_2_, which is an attractive material for photovoltaics,^100^ to materials of lesser abundance such as Zn_4_Sb_3_,^101^ which is among the most efficient of thermoelectric materials, and Cd_3_As_2_,^102^ which was recently identified as one of the few known examples of a three‐dimensional topological Dirac semimetal. Less well studied, Zn_3_As_2_ is an earth abundant semiconductor with 1.0 eV band gap and the potential to realize high hole mobilities.^103^

**Figure 5:**
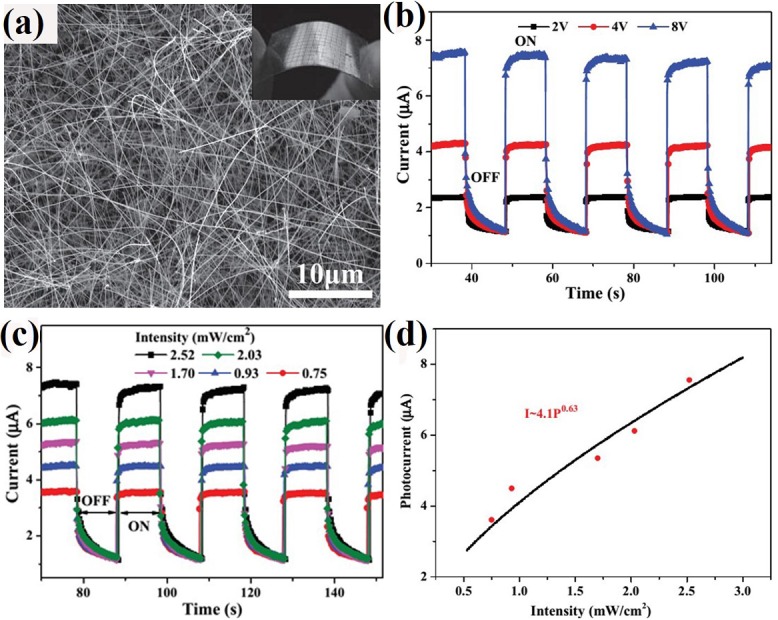
Flexible Zn_3_As_2_‐NW‐based PDs. a) SEM images; b) Photocurrent versus time plot of printed Zn_3_As_2_ NW‐array device on PET substrate using white light. c) Photocurrent versus time plot of printed Zn_3_As_2_ NW‐array device at different intensities. d) Photocurrent versus light intensity plot at a bias of 8 V. Reproduced with permission.^104^

Recently, single‐crystalline Zn_3_As_2_ NWs have been synthesized which are 100–240 nm in diameter, and hundreds of micrometers in length by a simple CVD method (**Figure** **5**a).^104^ Then FETs and visible‐light PDs are also fabricated and studied. The results showed that Zn_3_As_2_ NW revealed a typical p‐type characteristic with an effect hole mobility of 305.5 cm^2^ V^−1^ s^−1^. The photoresponse of the flexible device has also been investigated. As shown in Figure 5b is the current–time (*I–T*) curves of the flexible PD under a white light irradiation with different bias voltage of 2, 4, and 8 V. At the bias of 8 V, the dark current was 1.20 μA, then the photocurrent increased to 7.4μA as the white light illuminated. Compared with the rigid device, the flexible PD has a much lower photocurrent and dark current. It may be due to the worse contact between PET substrate and the NWs. Figure 5c shows the flexible PD with stable photocurrent‐switching property under various light intensties, revealing that the device has a good sensitivity even though the incident light has a slight variations. Fitting the measured date as shown in Figure 5d, *I* ≈ 4.1 *P* ^0.63^ was obtained, where *I* and *P* correspond to the photocurrent and the light intensity, respectively. Stable electrical characters of the flexible PD have also been studied. The result shows that there is no significant change of the conductance even after the flexible PD is bent by 30, 60, 90, and 120 cycles.

Besides Zn_3_As_2_ NWs, a series of other II–V compound‐NW‐based flexible visible PDs have also been fabricated including, Zn_3_P_2_,^105^ Cd_3_P_2_,^90^ and Cd_3_As_2_.^106^ These II–V NWs exhibited great photoresponse to the visible light due to their long minority carrier diffusion length, large optical absorption coefficient, and high carrier mobility. Meanwhile, flexible devices based on these materials also showed excellent flexibility and electrical stability. Through these researches we can see that II–V NWs have a potential application as next‐generation photoconductive materials to building blocks the flexible nano‐optoelectronic devices.

#### 2.2.3 Flexible Sulfide‐Based PDs

β‐In_2_S_3_ NW with a band gap of 2.0–2.3 eV has a potential application for high‐performance flexible visible PDs. as reported by Shen et al.^107^ The as‐prepared flexible PD showed an ultra‐high *I*_on_/*I*_dark_ ratio of up to 10^6^, which is six orders of magnitude higher than the best *I*_on_/*I*_dark_ values reported till now, and an excellent response to visible incident light with quantum efficiency and responsivity as high as 2.28 × 10^7^% and 7.35 × 10^4^ A W^−1^, respectively.

Some other kinds of sulfide NW based flexible PD have also been studied. For example, Chen et al. synthesized SnS nanoribbons with ca. 10–20 nm in thickness by the polyol refluxing process, which were used to fabricate flexible visible PDs with excellent photoresponse properties.^108^

**Figure 6:**
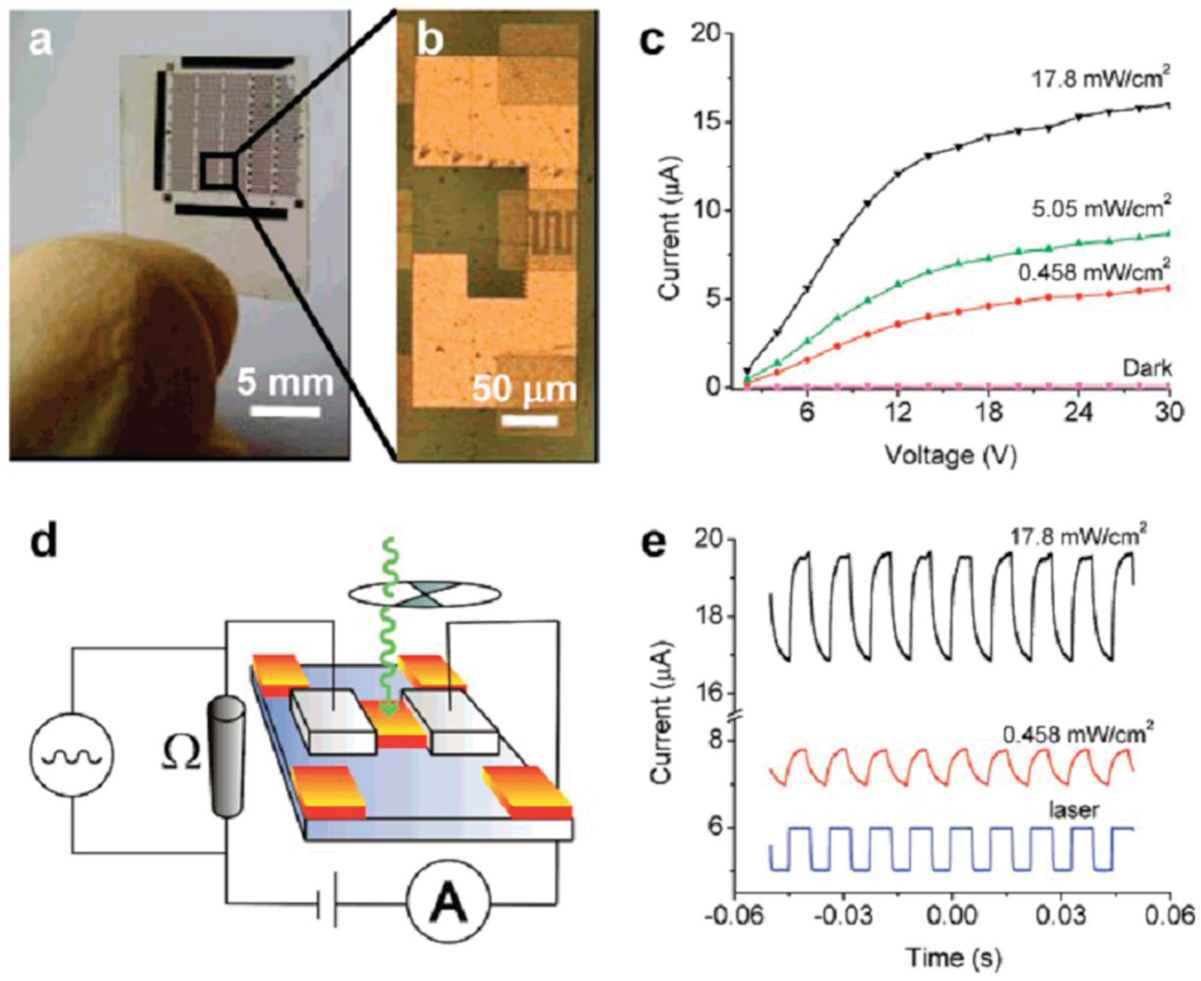
Arrays of PD devices based on CdS and characterization of the photoconducting properties. a) An array of devices on the transparent flexible PET substrate. b) An optical micrograph of a single PD device. c) Dark and photocurrents at different irradiation power densities. d) Schematic diagram for the time‐dependent signal current measurement. e) On–off switching using 514‐nm laser chopped at 90 Hz. Reproduced with permission.^109^

Very recently, Amos et al. introduced a universal and inexpensive way to fabricate CdS nanobelts onto a flexible substrate by utilizing the UV photooxidation patterned to directly induce the nucleation and growth from aqueous solutions (shown in **Figure** **6**).^109^ This technology is highly compatible with polymer substrates due to benign solvents utilized in the preparation of semiconductor and the absence of high temperature processing. As the flexible device was exposed under 514 nm light irradiated, it possessed an excellent photoresponse with the detectivity of 3 × 10^11^ cm Hz^1/2^ W^−1^ at a light frequency of 90 Hz.

### 2.3 Flexible Infrared PDs

Compared with the other types of PDs, infrared PDs have great importance in numerous civilian and military applications, including heat capacity mapping, thermal remote sensing, target tracking and environment monitoring.^110^, ^111^, ^112^, ^113^ Common materials for infrared PDs include GaSb,^114^ GaAs,^115^ InAs,^116^ and HgCdTe.^117^ Recent years, 1D nanostructures are also used in infrared PDs. For example, Lu et al. reported InAs NW PDs with a detection wavelength about ≈1.5 μm at room temperature with a photoresponsivity of 5.3 × 10^3^ AW^−1^.^118^ In order to avoid the negative effects of surface defect states and atmospheric molecules, the author introduced a half‐wrapped top‐gate by using 10 nm HfO_2_ as the top‐gate dielectric.

Till now, there are only a few examples could be found on flexible infrared PDs with 1D inorganic nanostructures as the sensing materials. Recently, single GaSb NW based PDs were fabricated on PET substrates, which exhibited high responsivity, fast‐response, and long‐term stability in photoswitching over a broad spectral range from ultraviolet to near infrared. Due to the much lower dark current on PET, the device has lower noise equivalent power of 2.0 × 10^−12^ W Hz^−1/2^.^119^ Although this flexible device showed great photoresponse properties and good flexibilities, the detective range was still less than 800 nm which limited its use as an infrared PD.

**Figure 7:**
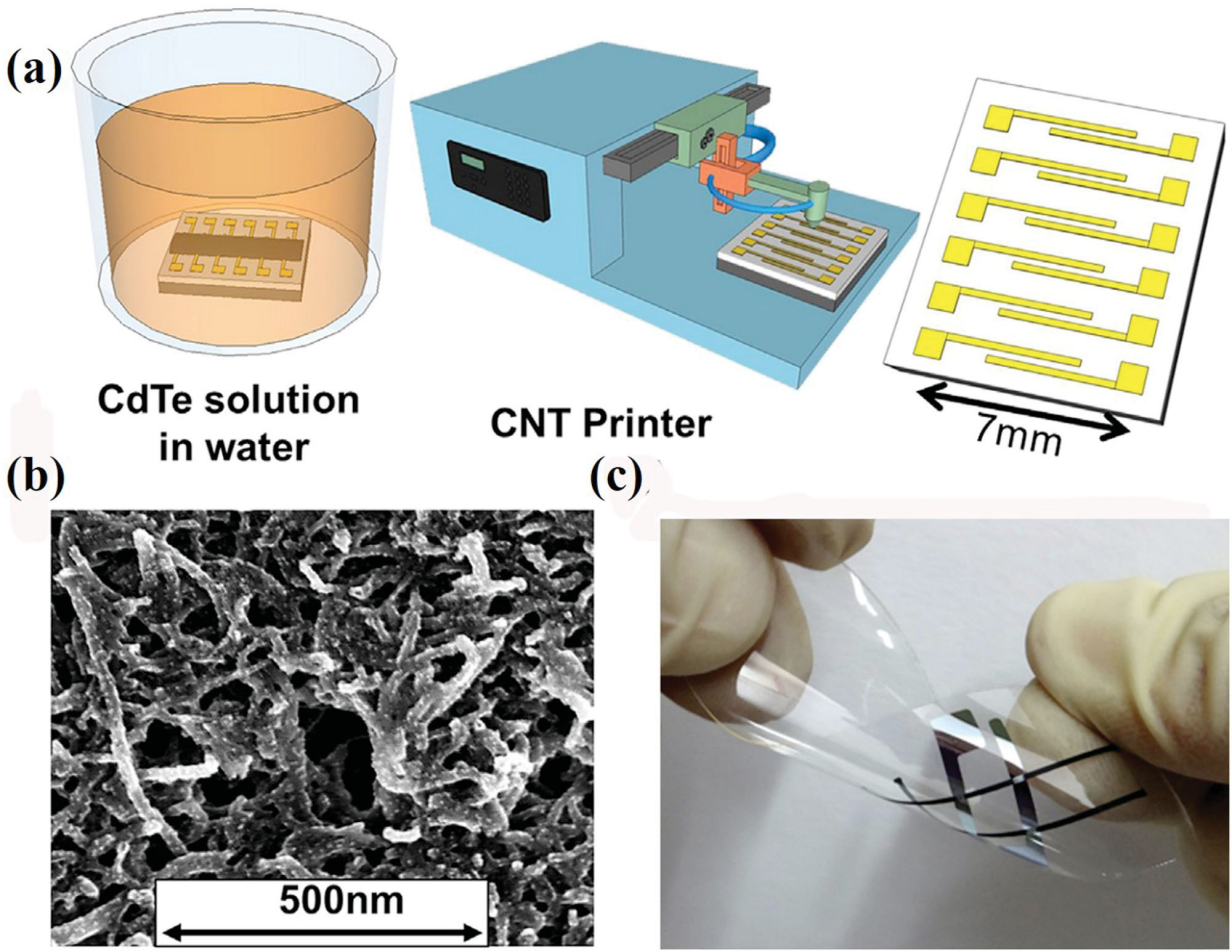
Flexible CNT based infrared PDs. a) Schematic drawing of the full detector. b) High resolution SEM view of the full printed CNT channel. c) inkjet‐printed sensor of the fabricated MWCNT‐NP's on a flexible transparent substrate. Reproduced with permission.^121^ Copyright 2014, Elsevier.

Carbon nanotubes (CNTs) have been extensively studied as electronic, photoelectronic, and piezoresistance materials.^120^ With a wide absorption light wavelengths of 354–2480 nm, PDs based on CNTs can be used to detect near‐infrared light. However, it still remains a challenge to achieve flexible CNT‐based infrared PD. Recently, Paltiel presented a simple wet chemistry technology for inkjet printing flexible PDs based on CNTs and CdTe nanocrystals (shown in **Figure** **7**).^121^ Figure 7a is a schematic drawing of the fabrication process for multi‐walled CNTs (MWCNTs)‐nanocrystals assembly. In this process, the MWCNTs lines are printed between a net of metal contacts and the CdTe NCs are adsorbed on top of the CNT lines. A typical high resolution SEM view of the printed CNT line with the adsorbed NCs is shown in Figure 7b. The as‐fabricated device has an obvious response to 980 nm infrared light at room temperature, with a fast response and recovery speed. Figure 7c shows the photograph of the flexible transparent device with excellent flexibility.

## 3 Flexible Organic–Inorganic‐Hybrid‐Based PDs

Organic–inorganic hybrid PDs have not only the advantages of inorganic based devices such as the broad‐band absorption and the excellent intrinsic carrier mobilities, but also the features of organic based devices, including tunable functionality and easy‐formation properties.^122^, ^123^, ^124^ In addition, the reaction at the interface between the organic polymers and inorganic semiconductors is good for the photoresponse properties.^125^, ^126^, ^127^, ^128^ In recent years, 1D organic–inorganic hybrid nanostructures have been widely investigated, which usually exhibited superior rectification, light‐emitting, and photovoltaic behaviors.^129^, ^130^, ^131^, ^132^, ^133^ However, the inorganic components in these hybrids are either quantum dots or nanoparticles. And devices with 1D inorganic semiconductor nanostructures as the inorganic component are rarely studied.

**Figure 8:**
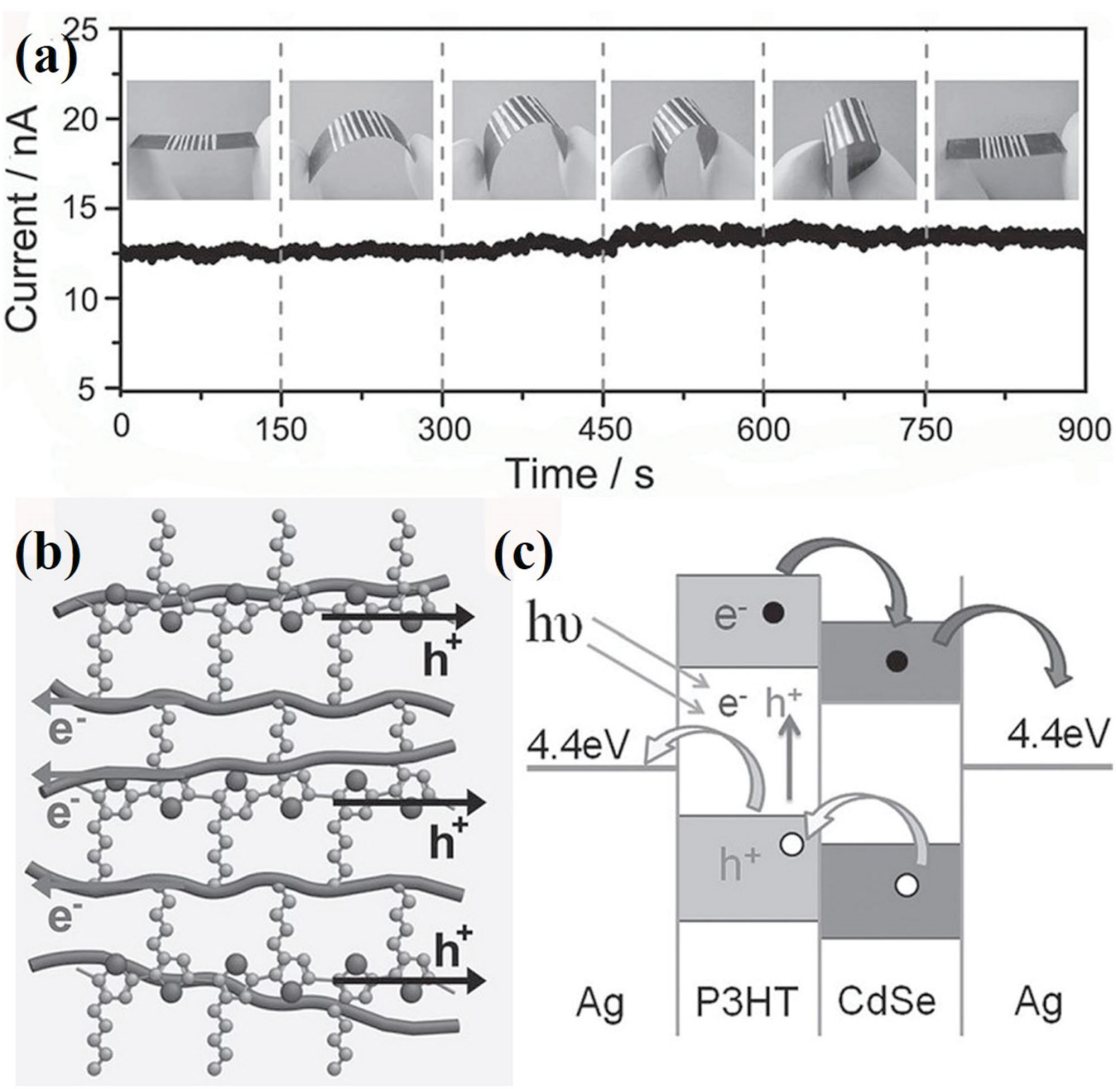
Flexible P3HT:CdSe NW hybrid based visible light PDs. a) The *I–T* curves when bent with different curvatures under a bias voltage of 3.0 V. b) Schematic of the hybrid film. c) Energy level diagram for P3HT and CdSe NWs under illumination. Reproduced with permission.^134^

We developed the first flexible hybrid PDs with P3HT and CdSe NWs as the components.^134^ P3HT was chosen as the organic component because it has great absorption in the visible range and high hole transport rate.^135^ CdSe NWs are utilized as the inorganic part because of its excellent electrical conductivity and caontrollable surface charge.^136^ Moreover, both the CdSe and P3HT can adsorb the spectra in the visible spectrum. **Figure** **8**a illustrates the mechanical flexibility of the hybrid PD under different bending conditions. The P3HT:CdSe NW hybrid device shows fast response properties with the rise and decay times measured on the millisecond timescale at high‐frequency light signals of 50 Hz. The probable enhancing mechanism for the hybrid PD has also been investigated. The interface of the hybrid film is a key factor in charge dissociation and transportation. When the photo‐carriers are generated, the electrons transfer to the material with higher electron affinity; in contrast, the holes transfer to the lower ionization potential. As shown in Figure 8b, in such a hybrid system, CdSe NWs could have a large interfacial area by combining with conjugated P3HT. The schematic energy‐level diagram of the hydrid films is shown in Figure 8c, we can see that P3HT are utilized as the hole‐transport material, while CdSe NWs is an effective electronic‐transport material, indicating the superlative photocurrent revealed in hybrid PDs. Furthermore, a 3D interconnected network in the hybrid film can be formed as CdSe NWs dispersed in the P3HT matrix, resulting in a large interface area for charge separation. Hence, good carrier transport and long‐lived charge separation could be obtained in the hybrid PD. These effects led to an impressive improvement of photoresponse of the hybrid PD compared with the single‐component devices. Similar results were also reported in other hybrid systems, such as the n‐type phenyl‐C61‐butyric acid methyl ester (PCBM)–p‐type Cd_3_P_2_ NWs system,^90^ and the n‐type PCBM–p‐type GaP NW‐ based, hybrid flexible PDs.^137^

**Figure 9:**
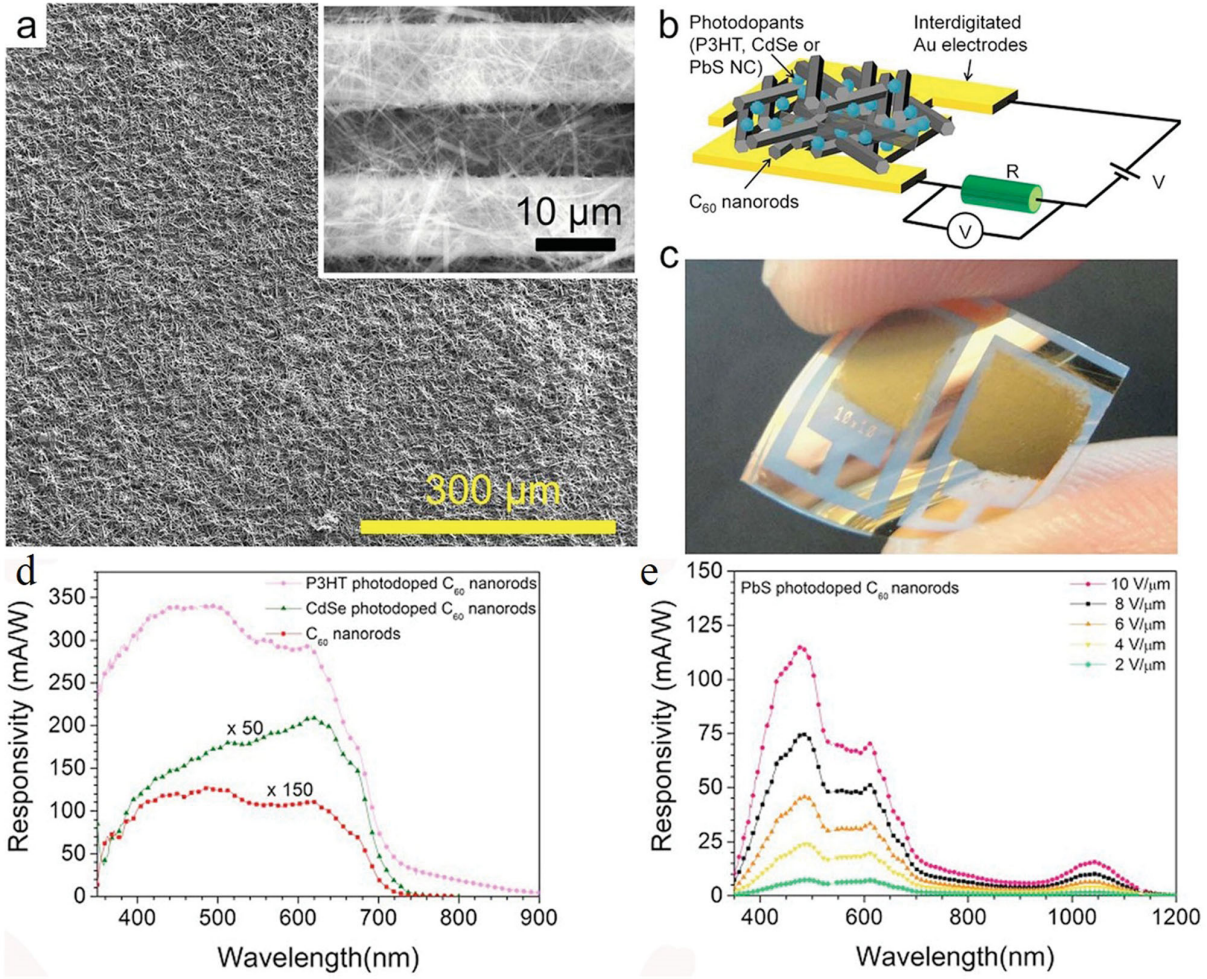
a) SEM image of a typical C60 nanorod photoconductor film. b) A schematic depicting the planar device architecture. c) Photograph of two typical C60 nanorod photoconductor devices fabricated on a flexible PEN substrate. d) Relative comparison of spectral responsivity obtained for C60 nanorod only and P3HT or CdSe NC photodoped C60 nanorod devices. e) Electric field dependent spectral responsivity of PbS NC photodoped device. Reproduced with permission.^138^ Copyright 2015, Macmillan Publishers Ltd.

The above studies exhibited good performance of the hybrid PDs, but the photoresponse properties can still be improved by changing the kind of organic and inorganic materials. Curry and his team reported that the photoresponse of the C60‐nanorod‐based PD could be improved 400 times due to an ultralow photodoping mechanism with a detectivity of >10^9^ Jones, a rise time 60 μs, and a linear dynamic range of 80 dB, which can measure 250 kHz AC signals.^138^ **Figure** **9**a shows a typical SEM image of the C60 nanorod film of more than a dozen micrometers in length and hundreds of nanometers in diameter. Figure 9b,c shows a schematic illustration of the PD and a photograph of the C60‐nanorod‐based flexible device. It can be seen that the device has a good flexibility. The organic TCNQ, R6G, P3HT and inorganic semiconductor PbS and CdSe NCs have been investigated to study the effect of photodoping on C60 nanorods. Figure 9d,e are the optical absorption spectrum of the photodopants. From Figure 9d, it is revealed that the use of P3HT and CdSe NCs as the photodopant provides 400‐ and 50‐fold improvement, respectively. Figure 9e shows the spectral responsivity of the PD based on PbS NCs photodoped at different wavelength and applied electric field. The devices also show a great enhancement in sensitivity, meanwhile they also display broadband UV–vis–NIR photosensitivity (350 to 1150 nm). These enhancements are achieved due to the ultralow photodoping mechanism. Some other works focused on the organic–inorganic hybrid PDs have also been reported, but most of them have not applied them in flexible devices.

## 4 1D‐Nanostructure‐Array‐Based Flexible PDs

1D nanostructure arrays have become an important subject for electronic and optoelectronic applications.^139^ To achieve the expected performance, 1D nanostructures with good alignment and precise patterning have been widely needed by most electronic applications. In consequence, more and more has been devoted to developing the alignment/patterning of inorganic 1D nanostructures. Recently, several techniques such as the bubble blown method,^140^ contact printing,^141^ Languir–Blodgett (LB) technique,^142^ electric/magnetic field alignment,^143^ and microfluidic‐assisted NW alignment,^144^ have been investigated. Compared with the single 1D nanostructure based PD, the aligned 1D nanostructure array is an efficient and simple remedy for low‐level photocurrents. Furthermore, compared with the random NW network, there is no contact‐potential barrier among the 1D nanostructure arrays which is beneficial for electronic transmission. The capability of assembling/printing different 1D nanostructures with tunable atomic composition on flexible substrates is very important in the field of flexible electronic with a broad spectrum. In the following, two types of NW array based flexible PDs are introduced: direct growth of aligned NWs and contact printing aligned NWs.

### 4.1 Direct Growth of NW Arrays on Flexible Substrates

**Figure 10:**
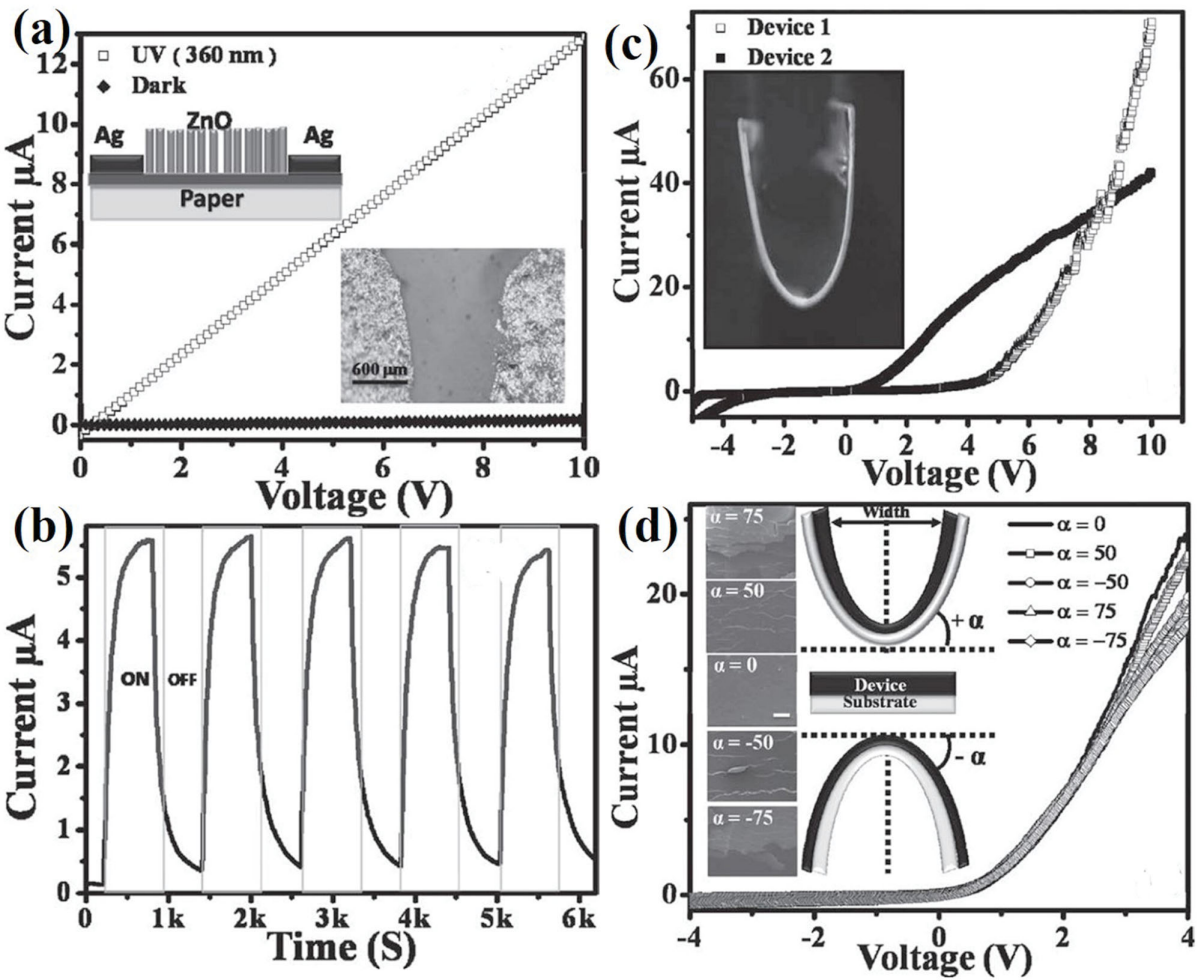
Flexible paper PD based on ZnO NW arrays. a) *I–V* plot shows the dark current and photocurrent with 360 nm illuminating UV light. b) The plot demonstrates the photoresponse at 5 V bias voltage. c) *I–V* characteristics of different p–n junction diode device structures. d) Electrical properties measured at different bending angles. Reproduced with permission.^145^

Selecting a suitable substrate is an important issue for development of both the method of direct growth of 1D nanostructure arrays and the structure of device design. For soft/flexible substrates, this is a difficult task, because high‐quality and aligned 1D nanostructures typically require specifically oriented, smooth substrates, as well as treatment with extreme temperatures. Among flexible substrates, paper with extreme surface roughness and thermal lability displays significant engineering challenges for the realization of 1D nanostructure growth. Chen et al. reported the controlled growth of aligned single‐crystal ZnO NWs on an economic and green paper substrate with a nonhazardous chemical solution and low temperature process.^145^ The innovation of this work is to regulate the paper's surface properties to control its conducting and semiconducting. The schematic of the paper PD is shown inset of **Figure** **10**a. The typical *I–V* properties of the PDs under continuous UV light illumination and in the dark were also shown. Figure 10b shows the photoresponse switching behavior of the PD. By modulating UV exposure, the photocurrent can be reproducibly switched with sensitivity of 60 at a low bias voltage of 5 V. Stable electrical performance at various twisting or bending conditions is an important factor for flexible substrates. Systematic analysis of the mechanical stability was carried out by controlling the PD to a specific bending angle and width. The results revealed that the flexible paper PD has a tiny change in the turn‐on voltage with increasing of bending angle because there are small traps formed between the NWs and the electrodes during bending.

Aside from paper, NW arrays can also be grown on other flexible substrates. For instance, ZnO NW array is grown on Kevlar fiber via the CVD method.^146^ Fiber‐based flexible UV PDs are then fabricated with these NW arrays. To speed up the UV response, P‐type conductive polymer poly (3,4‐ethylenedioxythiophene)/poly(styrenesulfonate) (PEDOT/PSS) is utilized to generate the internal electric field with ZnO NWs. Under UV off, Schottky type *I–V* curves demonstrate the formation of internal electric field. To measure the flexibility of the fiber‐based UV PD, the device was bent into a “U” shape. Even under such bending conditions, it still has a photosensitivity to UV light which means that the device has a very good flexibility. Such NW arrays grown on flexible polymer fiber used for PDs has also been reported by Bayindir.^147^

**Figure 11:**
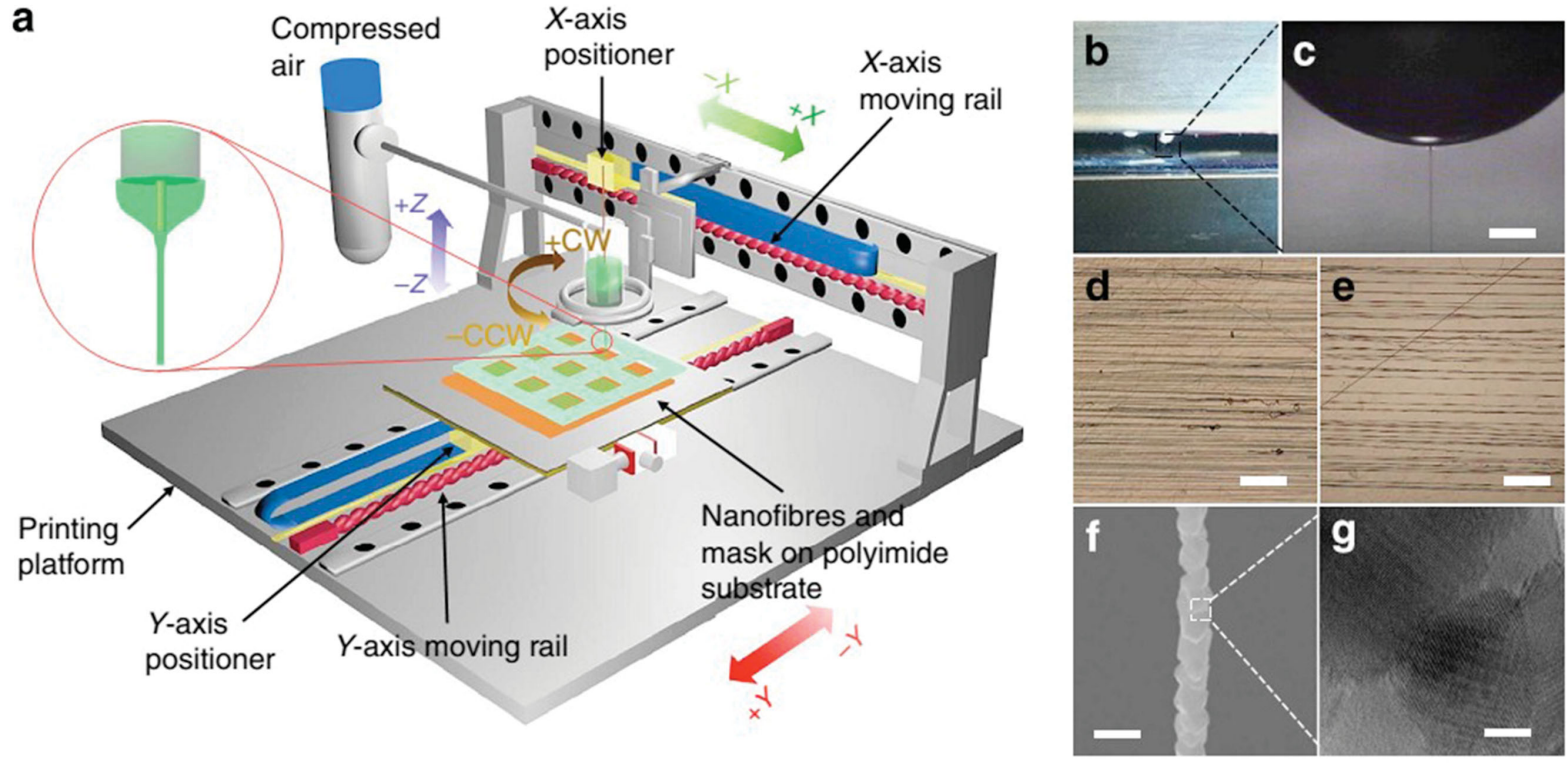
Schematic of two‐step all‐printable process and materials characterization. a) Printing setup schematic. b,c) Electrospinning ejection from the Taylor cone apex. d,e) Optical images of the as‐printed electrospun ZnAc/PVA nanofibers. f) SEM image of an as‐calcinated ZnO granular nanowire (GNW). Scale bar, 200 nm. g) TEM image of a GNW. Reproduced with permission.^148^ Copyright 2014, Macmillan Publishers Ltd.

Some other methods to grow 1D nanostructure arrays on flexible substrates were also studied. Electrospinning is a typical method to synthesize inorganic NWs and nanofibers which can spin the NWs directly onto the substrate. However, most of the electrospun NWs are disorderly and have rarely been employed for PDs. Recently, Fan and his group introduce an all‐printable fabrication technique to uniquely produce parallel ZnO NWs arrays onto a flexible substrate via the near‐field electrospinning technique, as shown in **Figure** **11**.^148^ Electrodes with a spacing of 2 μm were ink‐jet printed onto the NWs to investigate the optoelectronic properties. The device showed the highest detectivity and responsivity among the previous reports for NW‐based devices. The exciting results in this research can be used as a guideline utilizing other system materials to design high performance PDs.

### 4.2 Contact‐Printing of Aligned NWs on Flexible Substrates

**Figure 12:**
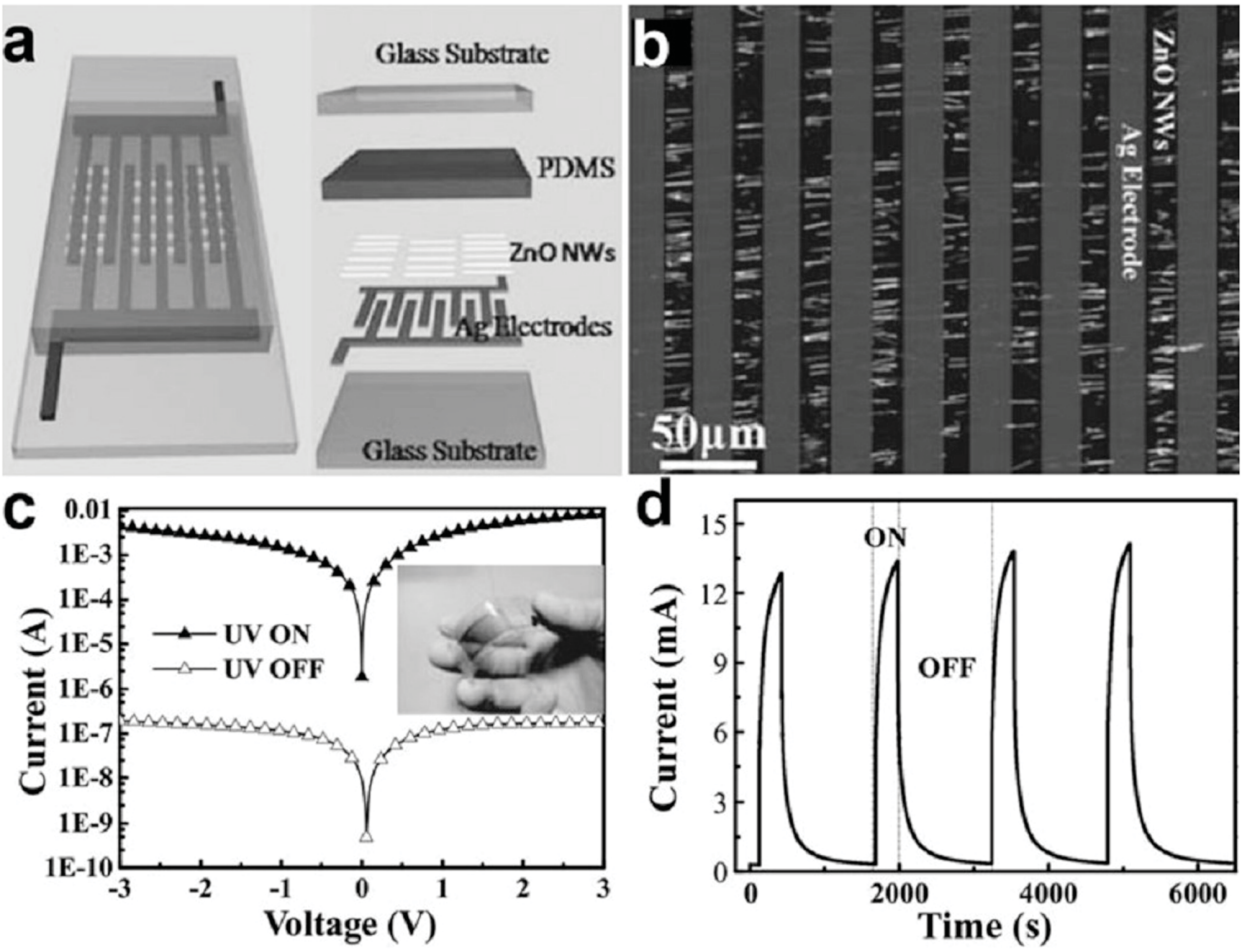
Flexible ZnO NW arrays based UV PD. a) Schematic design of an integrated UV sensor. b) Optical image of ZnO NWs connected in parallel between Ag electrodes. c, d) *I–V* curves and time‐dependent photoresponse of the flexible UV sensor with/without UV illumination, respectively. Inset of c) is the optical image of a flexible integrated NW UV sensor. Reproduced with permission.^155^

The contact‐printing method has proved to be an simple, and efficient way to assemble high‐density 1D nanostructure arrays on a large scale.^149^, ^150^, ^151^ This method can transfer the NWs to the receiver substrates to align the disorderly NWs into parallel arrays with a wide range of applications in electronic fields.^151^, ^152^, ^153^, ^154^ Flexible PDs based on NW array fabricated by the contact printing method were reported by several groups. Bai et al. reported an integrated ZnO NW UV flexible PDs at the macroscopic scale.^155^ **Figure** **12**a,b show a schematic diagram and SEM image of the device with Ag electrodes pressed onto the aligned NWs. As shown in Figure 12c, the *I–V* measurement confirmed the good Ohmic contact between the ZnO NWs and the Ag electrodes. The results show that as the device is exposed under UV radiation, the photocurrent has a great increased compared with the dark current. The *I–T* property of the device at a 4.5 mW cm^−2^ UV irradiance further confirm the ultrahigh on/off ratio which indicates the capacity to detect weak UV light. Compared to the individual NW devices, NW arrays devices have a great enhancement of photoresponse performance.

Similar improvement of photoresponse was also observed in InP NW^156^ and Zn_3_P_2_ NW^157^ arrays, as well as in ternary oxide NW arrays.^158^ All the fabricated flexible devices exhibited good flexibility, and electrical stability. These merits demonstrate that the contact printing is a good method for future fabrication of electronic and optoelectronic nanodevice.

## 5 New Concepts in Flexible PD Design with 1D Inorganic Nanostructures

Next‐generation electronic devices designed with a compact integration and small scale have been of widespread interest for scientists from different research areas. Nanoscale PDs, as one of the most widely used electronic devices, have shown great improvement from the fast development of nanotechnologies and nano‐science. Among the efforts devoted to this enhancement, some new concepts for flexible PDs based on 1D nanostructure are discussed in this section.

### 5.1 Piezo‐Phototronic PDs

**Figure 13:**
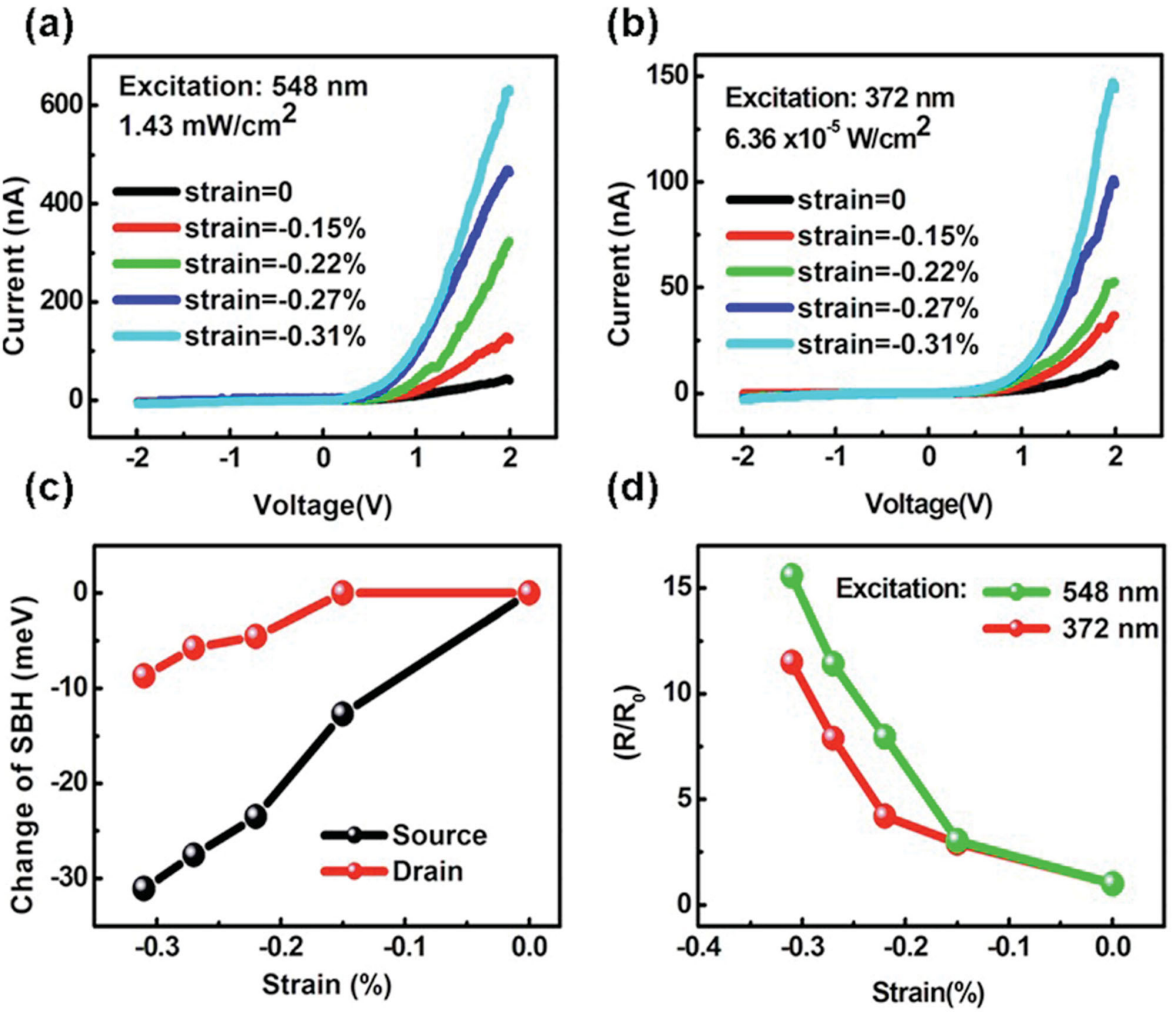
Flexible ZnO–CdS core‐shell NW based piezo‐potential enhanced PD. a,b) Typical *I–V* characteristics of a single ZnO–CdS wire‐based device under different compressive strains, excited by green light centered at 548 nm and UV light centered at 372 nm. c) The derived change in SBH as a function of compressive strains using the thermionic emission diffusion model. d) The change of responsivity under compressive strains, excited by green light centered at 548 nm and UV light centered at 372 nm. Reproduced with permission.^162^ Copyright 2012, American Chemical Society.

The piezo‐phototronic effect, which exists in wurtzite structure materials such as CdS, GaN and ZnO, has great potential in optoelectronic devices. Using strain, the properties of charge transport and recombination process can be regulated at the metal–semiconductor interface or the p–n junction. Previous reports have discussed more fundamental principles of this effect in detail. 1D nanostructures could be very useful in the preparation of strain controlled piezoelectronic devices where the deformation can be controlled by implementing the device on a flexible substrate. The piezo‐phototronic effect combined with flexible optoelectronics technology is used applied in solar cells, light‐emitting diodes, PDs etc. for enhancing the performance of the device.^159^, ^160^, ^161^ Recently, Wang et al.^162^ prepared a piezo‐potential enhanced PD based on the ZnO–CdS core–shell NW, and then proposed a theoretical model. Strain induced piezo‐potential can further enhance the performance of the PD by modulation of the Schottky barrier heights (SBHs) at the source and drain contacts. They illustrated how the SBHs and photocurrent of the PD under illumination have been affects by the piezoelectric polarization. Moreover, when the PD is set up to a –0.31% compressive strain the photoresponse of the ZnO–CdS device can be further improved for 10 times due to the piezo‐phototronic effect (**Figure** **13**). In addition, Liu et al.^163^ found that, at a low light intensity, the piezo‐phototronic effect greatly enhances the sensitivity for weak light detection. But as the light intensity increased, the SBHs got lower which gave the *I–V* curve a similar form to that of the Ohmic contact, so that the contribution of piezo charges had a weak influence compared with that of in low‐power illumination.

### 5.2 Stretchable PDs

**Figure 14:**
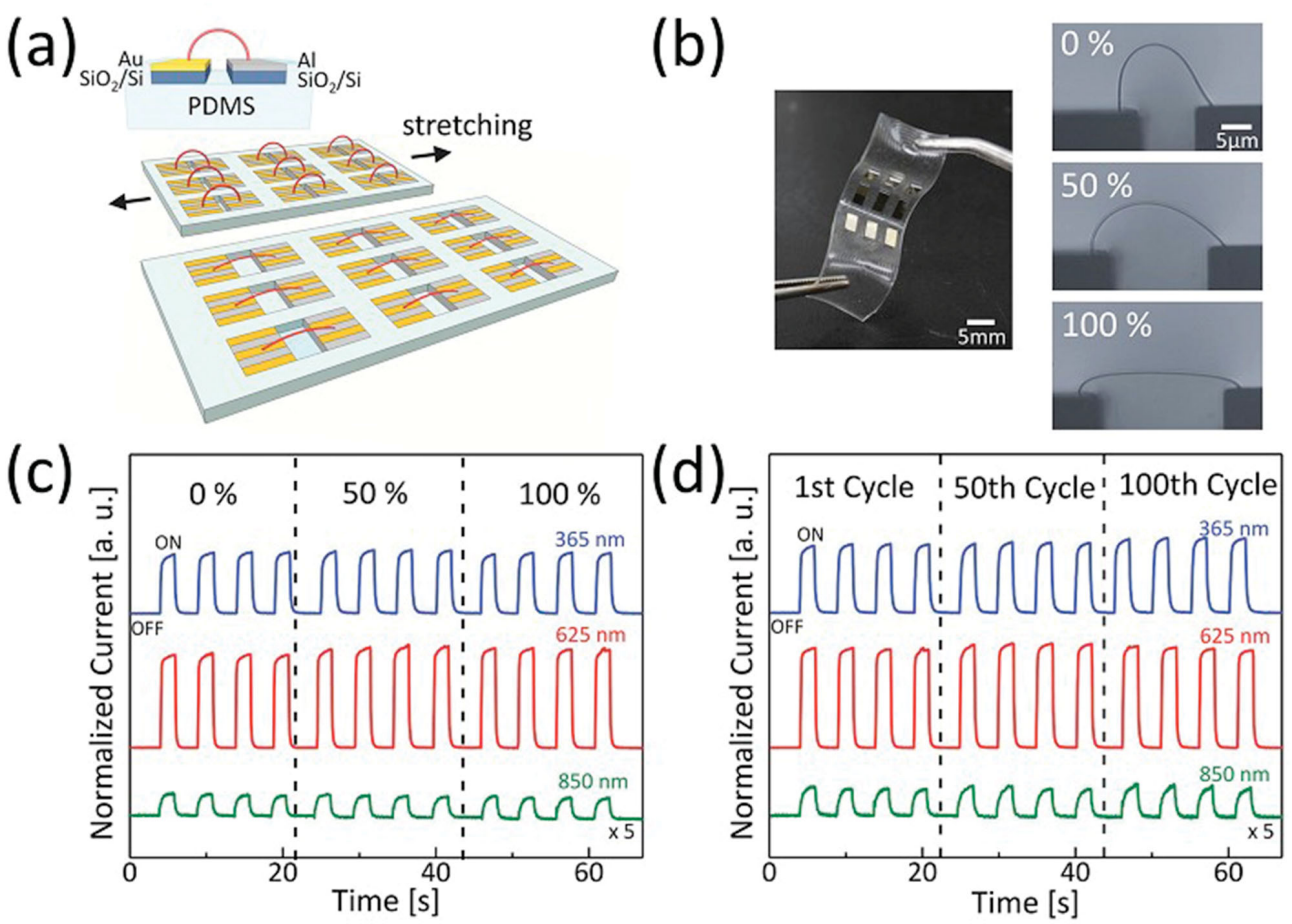
Stretchable UV–vis–NIR NWPD array. a) Schematic of a stretchable NWPD array of single hybrid NW arches. b) A photograph of the NWPD array and a series of optical microscopy images showing stretching of up to 100%. c,d) The photoresponse of the NWPDs during c) stretching of up to 100% and d) repeated stretching of up to 100 cycles at 100% for the UV–vis to NIR range. Reproduced with permission.^172^

Stretchable electronic devices have great advances for implantable devices in the human body.^164^, ^165^, ^166^ Stretchable devices, such as pressure sensors, gas sensors and PDs, could also be used in industry, bio‐organs, and under harsh environments.^167^, ^168^, ^169^, ^170^ Among these applications, stretchable PDs could be used to convert light as optical signal into an electrical signal. Stretchable PDs can be integrated with biological systems including electronic eye cameras, wearable monitoring devices as well as many other applications. However, very few reports have been focused on 1D nanostructure based stretchable PDs. Polydimethylsiloxane (PDMS) with high stretchability, excellent optical transparency and good biocompatibility is one of the most applied substrate for stretchable devices. Lee and his group first introduced a lithographic filtration technology to manufacture embedded NWs based stretchable PDs.^171^ The PDs keep their functionalities even as they are stretched up to 100% condition. However, the *I*_on_/*I*_off_ ratio decreased when the device stretched with a slower response and recover rate, due to the NW‐polymer chain interactions considering the low oxygen content and slow gas diffusion rate. Then, Je et al. employed high‐performance, stretchable UV–vis–NIR NWPDs by a direct‐writing, meniscus‐guided method.^172^ They employed an effective method to grow NW arches between two electrodes on PDMS substrates as schematically illustrated in **Figure** **14**a. The NWPD array exhibited superior stretch ability (up to 100%) and flexibility, as obviously investigated by a photograph (Figure 14b). The photoresponse of the NWPDs was almost unchanged even under substantial or repeated stretching. In addition, under stretching of up to 100%, the on/off ratio and response time of the device were almost constant. These outstanding properties clearly display the great photoelectrical stability under ultra‐stretching states, because the NW arches with the stretchable architecture can relax the external strain. The direct writing method of fabrication of NW arches presents potential application for next‐generation stretchable and high‐performance photoelectronic devices.

### 5.3 Integrated Self‐Powered PDs

**Figure 15:**
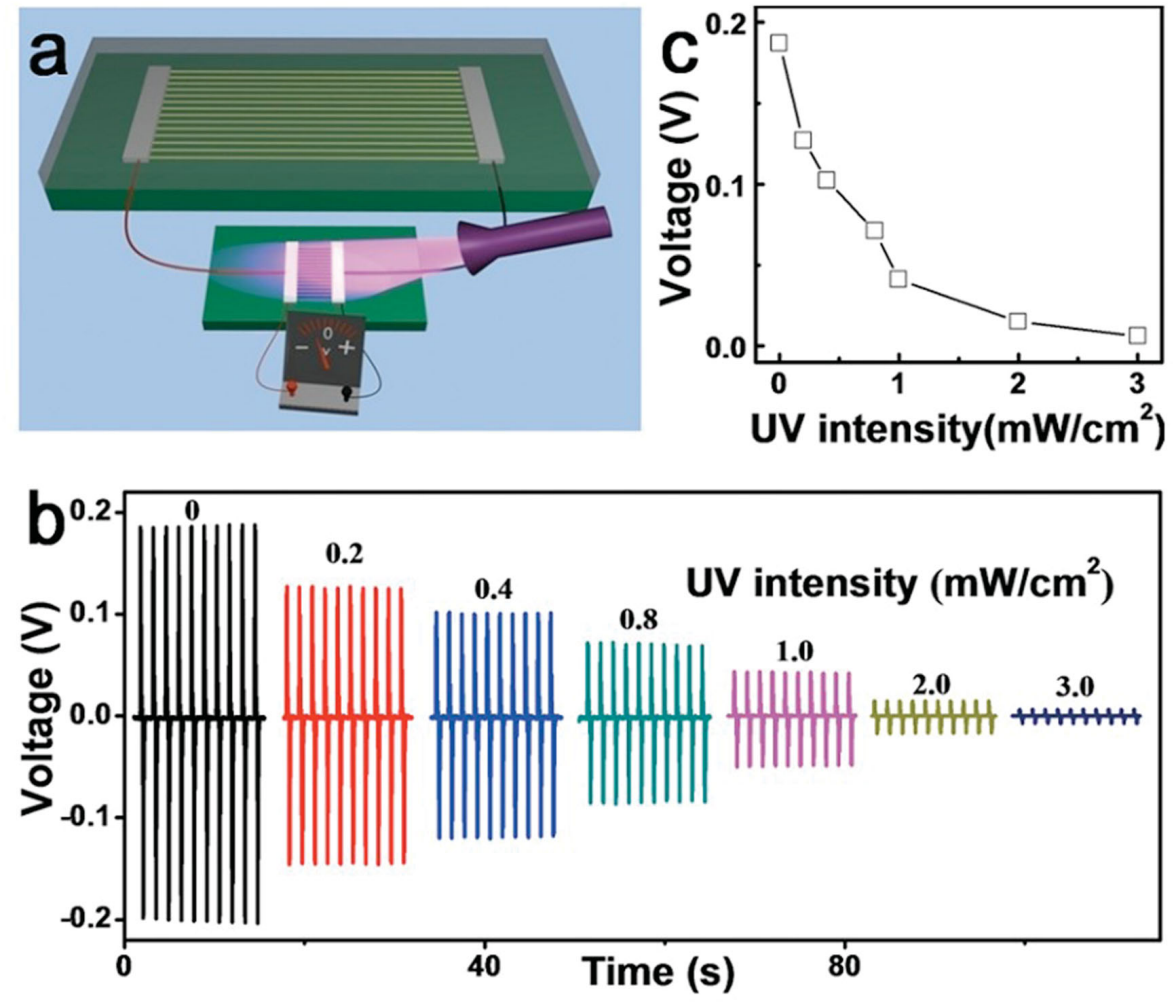
Flexible integrated ZnO NW based UV PD. a) Schematic of a self‐powered nanosystem composed of a UV sensor and FTNG. b) UV photoresponse of a ZnO NW UV sensor powered with a FTNG. c) Plot of the voltage drop on a UV sensor vs. UV light intensity. Reproduced with permission.^177^ Copyright 2012, American Chemical Society. Reprinted with permission from ref. 177 ©2012, American Chemical Society.

Integrated self‐powered nanosystem with power supplies and functional nanodevices can be used in various applications, such as medical therapy, environmental monitoring and optoelectronics.^173^, ^174^, ^175^ Integrated PDs received extensive attention since they may play an important role in the particular applications, including chemical and biosensing and wireless sensor networks.^176^ Due to the production of devices that do not require an external power supply, the extra weight of the system can be avoided. In general, this type of self‐powered PD is made up of a light sensor, an electrical measurement system, and a power unit. For instance, Wang et al. power a flexible integrated ZnO NW based UV PD by a flexible transparent nanogenerator (FTNG).^177^ **Figure** **15**a shows the schematic of a self‐powered nanosystem. The corresponding voltage drop is about 0.2 V on the UV PD with the large resistance, when the UV light is off. Since irradiated under the UV light, the carriers increase leading to the decreasing of the resistance. More carriers can be generated with the UV light intensity increased. Meanwhile the voltage drop decreases further. By monitoring the changes of the voltage on the device, UV light can be quantitatively detected. Therefore, a FTNG can be successfully used to drive the device to form a flexible self‐powered UV PD.

Our group also fabricated several kinds of self‐powered PD systems with different device structures. For example, CdSe NW PDs were integrated with a flexible all‐solid‐state GeSe_2_ supercapacitor to form a self‐ PD nanosystem without any external bias voltage.^178^ A self‐powered visible PD system driven by graphene microsupercapacitors was also recently designed.^179^ The optimized electrodes in the microsupercapacitor device were utilized as the source and drain electrodes of the CdS PD as well, configuring an integrated, self‐powered, visible light PD without an external power source. Compared to the time‐dependent photoresponse of the PD driven by an external power source, the integrated PD system showed similar performance, indicating the potential applications of the integrated self‐powered systems for next generation highly compacted, highly flexible and extremely light electronics.

Although integrated self‐powered PDs are generally planar, fiber‐based integrated PDs have also been studied recently. However, compared with the planar integrated self‐powered PDs which may have a restriction for the actual applications, a fiber‐based integrated PD may have its special advantages. Recently, a fiber‐based integrated self‐powered PD has been successfully fabricated.^180^ In this system, a microscale flexible asymmetric supercapacitor (SC) using Co_3_O_4_ NWs on titanium wire and graphene on carbon fibers electrodes was used as energy‐storage device. Meanwhile, a two‐dimensional graphene layer was used as the light‐sensitive material. The device shows an excellent photoresponse to white light. The photoresponse mechanism of the integrated system can be explained that when the device exposed to the light, the electron–hole pairs became separated under an external field supplied by the fully charged SC, leading to the increased leakage current of the SC. Hence, by monitoring the changes of the leakage current, photodetection can be accomplished. As a flexible integrated system, the performances of both the SC and PD changed little when the device was bent into different states, demonstrating the excellent flexibility of the integrated self‐powered PDs which combined a SC and a PD on a single fiber.

## 6 Summary and Outlook

In this review, the most recent developments on 1D, inorganic‐nanostructure‐based, flexible PDs have been presented. We have given a summary of the applications of inorganic 1D nanostructures and the performance of advanced flexible PDs fabricated using such nanomaterials as reported in the latest literature. The fascinating achievements to date should encourage more researchers to make additional efforts to tackle new challenges in the future.

To further improve the performance of nanostructured PDs, the properties of 1D nanostructures and the design of the flexible devices should be paid more attention in the future. Although the research on various 1D nanostructures has made remarkable progress and significant achievements, some problems still exist. Firstly, the effect of size heterogeneity on the performance is very obvious. So more precise control of morphology, hierarchical, crystallization, and orientation assembly is urgently needed. However, 1D nanostructures grown by bottom‐up processes still have challenges because the small dimensions have a tendency to aggregate. To address this problem, two effective approaches can be used. One is a novel 3D printing method used to print quality NWs or uniform NW arrays directly. The other is the top‐down approaches improving which has the advantage for large scale integration. Secondly, uniform 1D nanostructures still do not ensure the uniform physical and chemical properties, electronic transport properties etc. It is very important but still a challenge to minimize the variability NW‐to‐NW. Thirdly, for fabricating and integrating the scalable flexible PDs, the yield of 1D nanostructures is still significantly insufficient. Nowadays, there are more or less advantages or weaknesses on the 1D nanostructures assembly techniques. This situation will create some issues in printing 1D nanostructures accurately on flexible substrates. Hence, more suitable 1D nanostructure assembly methods need to be prepared to provide better quality of 1D nanostructures for flexible PDs.

Although significant progress about the design of flexible PDs has been made, there are still challenges and future directions to study. Firstly, compared with the classical PDs, flexible PDs based on 1D nanostructures are still at a primary stage. Because 1D‐nanostructure‐based flexible PDs have much lower properties in bandwidth and efficiency and the performance of between devices is variable; their progress and applications have been greatly confined. To solve these problems, we should develop new measurement and evaluation technologies. If an effective method can be employed to guarantee both outstanding flexibility and high performance for PDs, more commercial products based on 1D nanostructures will be developed. Secondly, the biggest challenging to put the nanoscale flexible PDs into commercial applications is their high cost. It is well known that most of the assembly of 1D nanostructures into flexible PDs still needs complex technology such as lithography processes with expensive instruments and time‐consuming. Hence, more attention should be focused on how to achieve low‐cost preparation of flexible PDs on a large scale. Printed electronics have the advantages of large scale, flexibility and low‐cost which may be good for fabricating large scale flexible PDs and needs more studies from researchers. At last, for future directions on flexible PDs based on 1D nanostructures, integrated systems with multi‐functional nanodevices will become a hot topic for next generation flexible nanodevices. If one can assemble other functional nanodevices with a PD into a nanosystem, a large variety of functions can be incorporated, and then it can be introduced to the market with great potential of commercial and industrial applications.
